# Boston bowel preparation scale score 6 has more missed lesions compared with 7–9

**DOI:** 10.1038/s41598-024-52244-8

**Published:** 2024-01-18

**Authors:** Jung Kim, Ji Min Choi, Jooyoung Lee, Yoo Min Han, Eun Hyo Jin, Joo Hyun Lim, Jung Ho Bae, Ji Yeon Seo

**Affiliations:** Department of Internal Medicine and Healthcare Research Institute, Healthcare System Gangnam Center, Seoul National University Hospital, Seoul, Korea

**Keywords:** Gastroenterology, Health care

## Abstract

Adequate bowel preparation is an important factor in high-quality colonoscopy. It is generally accepted that a Boston Bowel Preparation Scale (BBPS) score ≥ 6 is adequate, but some reports suggest ≥ 7. Subjects who underwent colonoscopy at least twice within 3 years from August 2015 to December 2019 were included. Polyp detection rates (PDRs), adenoma detection rates (ADRs), and number of polyps including adenomas were compared stratified by baseline colonoscopy (C1) BBPS score. Among 2352 subjects, 529 had BBPS 6 (group 1) and 1823 had BBPS 7–9 (group 2) at C1. There was no significant difference in PDR or ADR at C1 and follow-up colonoscopy (C2) between the two groups. However, the numbers of polyps (1.84 vs. 1.56, *P* = 0.001) and adenomas (1.02 vs. 0.88, *P* = 0.034) at C2 were significantly higher in group 1 than group 2, respectively. Segmental BBPS score 2 in group 1 compared to group 2, especially, showed higher PDR (*P* = 0.001) and ADR (*P* = 0.007) at C2. BBPS 6 is associated with a higher number of polyps and adenomas in short-term follow-up colonoscopy than BBPS 7–9. To reduce the risk of missed polyps, a thorough examination is necessary for BBPS 6.

## Introduction

Colonoscopy is an important modality that can reduce the incidence and mortality of colorectal cancer^1–3^. Adequate bowel preparation is essential for effective colonoscopy. Inadequate bowel preparation reduces the cecal intubation rate^4^ and polyp detection rate (PDR) and prolongs insertion times and withdrawal times^5^, and it is associated with patient dissatisfaction^6^. In addition, after colonoscopy with inadequate bowel preparation, the examination must be repeated at intervals shorter than the recommended interval^7^, which leads to an economic burden^8^. Crucially, inadequate bowel preparation is an important cause of interval cancer^9^. Therefore, according to the US Multi-Society Task Force on Colorectal Cancer guidelines, as a quality indicator of colonoscopy, a rate of adequate bowel preparation ≥ 85% is recommended^10,11^.

**Figure 1:**
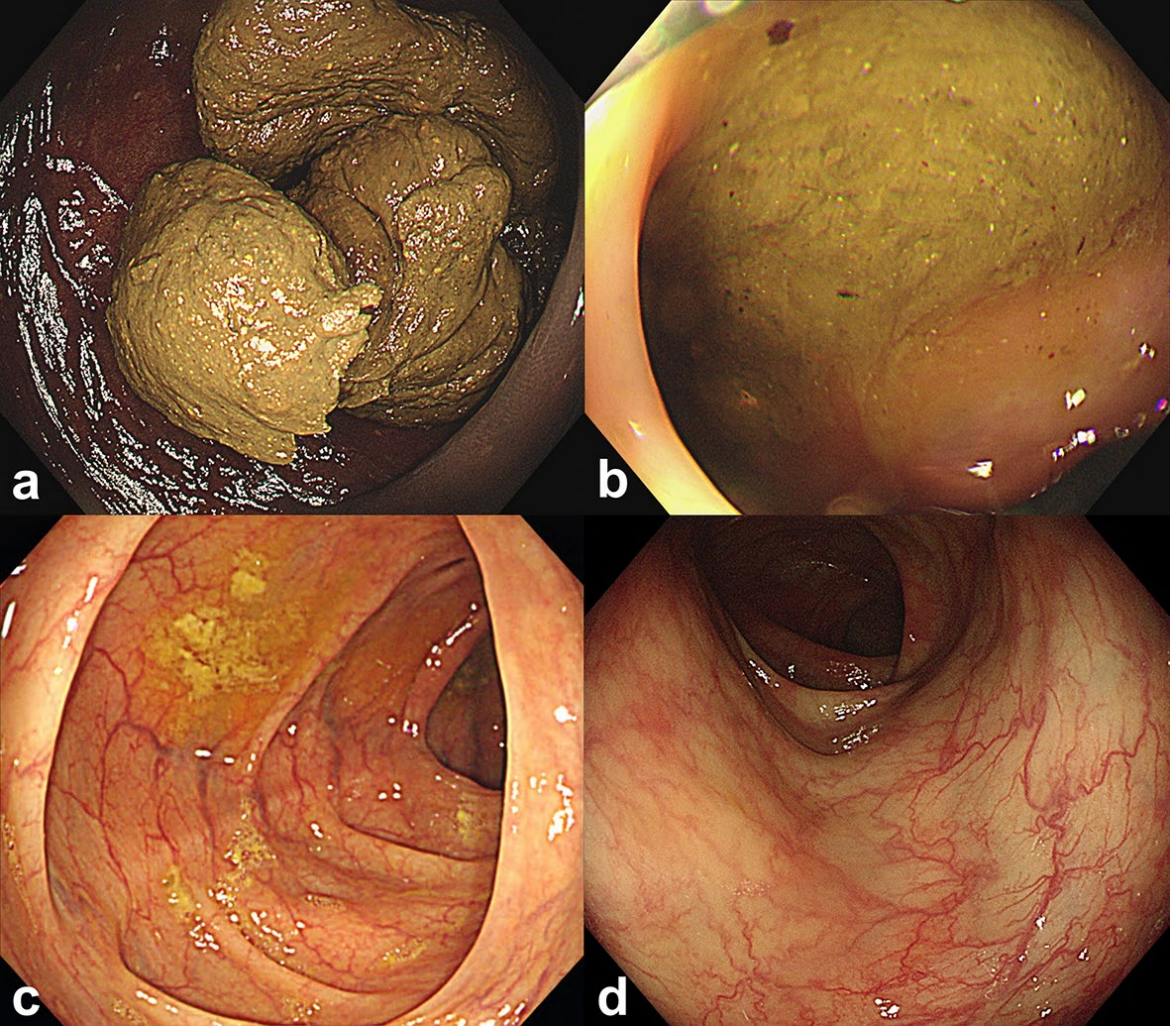
Examples of the BBPS. (**a**) Segment score 0, unprepared colon segment with mucosa not seen because of solid stool that cannot be cleared. (**b**) Segment score 1, portion of mucosa of the colon segment seen, but other areas of the colon segment not well seen because of staining, residual stool, and/or opaque liquid. (**c**) Segment score 2, minor amount of residual staining, small fragments of stool and/or opaque liquid, but mucosa of colon segment seen well. (**d**) Segment score 3, entire mucosa of colon segment seen well with no residual staining, small fragments of stool and/or opaque liquid.

To determine adequate bowel preparation, a well-validated scoring system is warranted. The Boston Bowel Preparation Scale (BBPS) has been widely used among various bowel preparation scoring tools since 2009^5,12^ (Fig. 1). BBPS is a 4-point scoring system (range, 0–3) applied to each of the three broad regions of the colon (right, transverse, and left colon); the points for each segment are summed for a total BBPS score (range, 0–9). The advantage of BBPS is that the score is evaluated after washing and suctioning of colonic contents, reflecting the extent of colonic mucosa actually observed^5^.

The recommended surveillance interval after colonoscopy is based on adequate bowel preparation^11,13,14^. However, the standards for adequate bowel preparation are controversial^11,14^. BBPS developers recommended that a score of less than 5 points corresponded to inadequate bowel preparation^5^. On the contrary, the European Society of Gastrointestinal Endoscopy (ESGE) defined less than 6 points as inadequate bowel preparation^14^. The American Society for Gastrointestinal Endoscopy (ASGE) defined visualization of polyps > 5 mm as adequate bowel preparation^11^. The ASGE and the ESGE recommend repeated examination within 1 year if a colonoscopy with inadequate bowel preparation is performed. The absence of clear criteria might cause increased medical cost and risk of complications due to overuse or increased cancer risk due to underuse. We aimed to compare the PDRs and adenoma detection rates (ADRs) at follow-up colonoscopy according to baseline BBPS scores. We focused on whether BBPS score 6 differs from BBPS score 7–9 in detection rates and missed lesions at follow-up colonoscopy.

## Methods

### Study design

**Figure 2:**
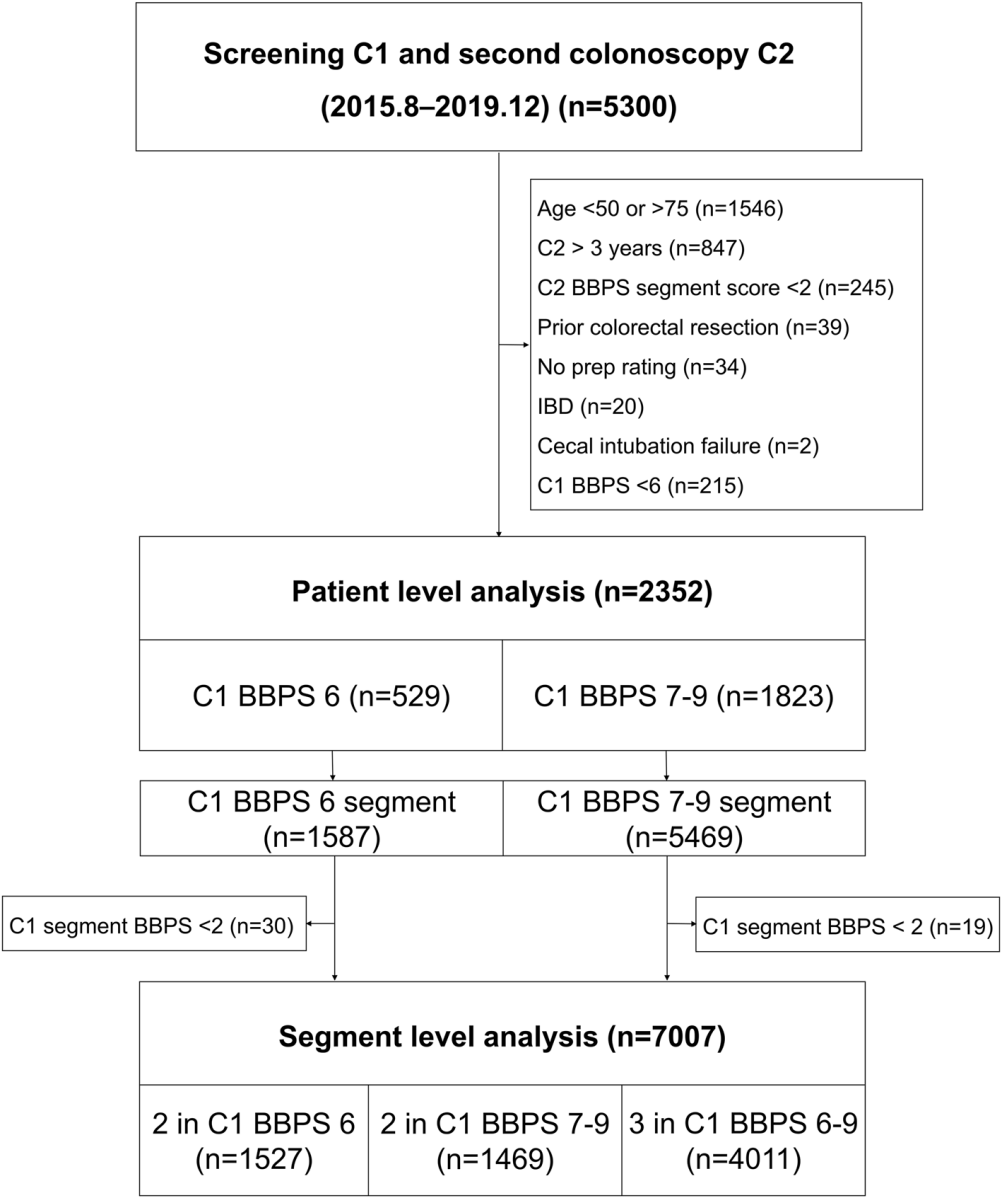
Flow diagram of the study. C1, baseline colonoscopy; C2, follow-up colonoscopy; BBPS, Boston Bowel Preparation Scale.

Participants between 50 and 75 years who underwent colonoscopy at least twice from August 2015 to December 2019 at Seoul National University Hospital Healthcare System Gangnam Center were retrospectively enrolled. The second colonoscopy had to be done within 3 years of the first colonoscopy to minimize the chance of de novo polyp based on previous studies^15,16^. Exclusion criteria were as follows: prior colorectal resection, history of inflammatory bowel disease, familial polyposis syndrome, inability to achieve cecal intubation, and subjects who had inadequate bowel preparation at second colonoscopy (BBPS segment scores of < 2) (Fig. 2). The study was approved by the institutional review board of Seoul National University Hospital (Institutional Research Board 2109-110-1254). It conformed to the ethical guidelines of the World Medical Association’s Declaration of Helsinki. Informed consent from individual participants was waived by Ethics Committee of Seoul National University Hospital, because this study used retrospectively collected data.

### Colonoscopy and bowel preparation

Colonoscopies were performed by 27 board-certified endoscopists who had performed at least 2000 cases. For colonoscopy, CF-H260 or CF-HQ290 series endoscope (Olympus, Tokyo, Japan) were used. All colonoscopists had equal chance to use HQ290 series. According to the endoscopist’s decision, NBI was freely used. For bowel preparation, a split-dose regimen of 2-L polyethylene glycol plus ascorbic acid (Coolprep; Taejoon Pharm, Seoul, Korea) was used^17^. Removed polyps were assessed by experienced board-certified pathologists. Immediately after the procedure, the endoscopist assessed the cleanliness of the colon using BBPS^5^. Endoscopists were trained until they had high concordance rates in bowel preparation scoring and surveillance interval^18^.

The baseline colonoscopy was denoted as C1, and the follow-up colonoscopy was denoted as C2. Cases with a BBPS segment score ≥ 2 for all 3 segments were included to assess the missed lesions of C1. We analyzed pairs of C1 and C2 colonoscopy at the patient level. We also analyzed 3 colon segment pairs (right, transverse, and left) of C1 and C2 colonoscopy at the segment level. Since the number of cases was too small to calculate detection rates in examinees with BBPS scores 0–5, statistical analysis was performed only for BBPS scores 6–9.

### Study outcomes

Subjects were classified into two groups according to C1 BBPS score 6 (group 1) and 7–9 (group 2). The primary outcome was the proportion of patients with polyps and adenomas at C2 stratified by C1 BBPS scores, defined as PDR and ADR, respectively. The secondary outcome was the mean number of polyps and adenomas at C2 according to C1 BBPS score. The numbers of polyps and adenomas were selected to compensate for the disadvantage of PDR and ADR, in which detection of one or more indicates the same value. Detection rates and numbers of adenomas > 5 mm (adenomas > 5 mm) and advanced adenomas (any of the following features: villous component, high-grade dysplasia, or ≥ 1 cm) were calculated as well. We also analyzed the proportion of segment pairs with polyps and adenomas at C2 stratified by C1 segment BBPS scores, which was defined as segmental PDR and ADR.

### Statistical analysis

Values are expressed as number (percentage) and mean ± standard deviation or median (interquartile range (IQR)). A chi-square test was used to evaluate categorical variables, and the Mann–Whitney U test and Kruskal Wallis test were used for comparison of nonparametric variables. Linear regression analysis was used to evaluate the risk of adenoma at C2. Multiple linear regression analysis was conducted using the significant outcomes determined by simple linear regression analysis. Statistical analysis was performed using SPSS version 25 (IBM, Armonk, NY, USA), and *P* < 0.05 was considered statistically significant.

## Results

### Baseline characteristics of the study population

46,569 colonoscopies were performed from August 2015 to December 2019 at Seoul National University Hospital Healthcare System Gangnam Center. 5300 subjects underwent colonoscopy at least twice within 3 years (Fig. 2). The baseline characteristics of the study participants are shown in Table 1. Among the 2352 subjects included in this study, 529 (22.5%) had BBPS score 6 and 1823 (77.5%) subjects had BBPS score 7–9. Overall, men were more frequent than women (72.5 vs. 27.5%). The median age was 58 (IQR 54–64), and median BMI was 23.9 (IQR 22.2–25.7). Most subjects underwent colonoscopy for surveillance (94.8%). The median follow-up duration was 727 days (IQR 401–909). There was no significant difference in baseline characteristics between the two groups.

**Table 1:** Baseline characteristics according to BBPS score.

	Total	C1 BBPS 6	C1 BBPS 7–9	*P* value
	N = 2352	N = 529	N = 1823	
Sex				
Male	1705 (72.5)	396 (74.9)	1309 (71.8)	0.166
Female	647 (27.5)	133 (25.1)	514 (28.2)	
Age (year)	58 (54–64)	59 (54–66)	58 (54–63)	0.163
BMI (kg/m^2^)	23.9 (22.2–25.7)	24.1 (22.4–26.0)	23.8 (22.2–25.6)	0.503
Purpose				
Screening	122 (5.2)	29 (5.5)	93 (5.1)	0.728
Surveillance	2230 (94.8)	500 (94.5)	1730 (94.9)	
Follow-up interval (d)	727 (401–909)	700 (385.5–861)	729 (412–923)	0.730

Values are expressed as n (%), or median (interquartile range).

BMI, Body mass index; BBPS, Boston bowel preparation scale; C1, baseline colonoscopy.

### Polyp and adenoma detection rates according to BBPS score

Detection rates of polyps, adenomas, and advanced adenomas (AADR) at C1 according to C1 BBPS score are displayed in Table 2. All factors did not show statistical significance between the two groups. In addition, detection rates and numbers of polyps, adenomas, adenomas > 5 mm, and advanced adenomas at C2 between the two groups were compared (Table 3 and supplementary Table 1). There was no significant difference in PDR, ADR, ADR > 5 mm, and AADR between the two groups. However, the numbers of polyps and adenomas were significantly higher in group 1 than group 2 (1.84 ± 1.89 vs. 1.56 ± 1.73, *P* = 0.001, and 1.02 ± 1.36 vs. 0.88 ± 1.27, *P* = 0.034, respectively). The numbers of adenomas > 5 mm and advanced adenomas were also slightly higher in group 1, but statistical significance was not met (*P* = 0.083 and *P* = 0.522, respectively).

**Table 2:** Polyp, adenoma, and advanced adenoma detection rates of baseline colonoscopy according to BBPS score.

C1 polyp detection	C1 BBPS		*P* value
	6	7–9	
	N = 529	N = 1823	
Polyp	471 (89.0)	1639 (89.9)	0.562
Adenoma	410 (77.5)	1386 (76.0)	0.482
Advanced adenoma	36 (6.8)	100 (5.5)	0.252

Values are expressed as n (%).

BBPS, Boston bowel preparation scale; C1, baseline colonoscopy; No, number.

**Table 3:** Polyp, adenoma, adenoma > 5 mm, and advanced adenoma detection rates of follow-up colonoscopy according to BBPS score.

C2 polyp detection	C1 BBPS		*P* value
	6	7–9	
	N = 529	N = 1823	
Polyp	385 (72.8)	1251 (68.6)	0.067
Adenoma	281 (53.1)	897 (49.2)	0.113
Adenoma > 5 mm	66 (12.5)	181 (9.9)	0.092
Advanced adenoma	16 (3.0)	47 (2.6)	0.576
Polyp No	1.84 ± 1.89	1.56 ± 1.73	0.001*
Adenoma No	1.02 ± 1.36	0.88 ± 1.27	0.034*
Adenoma > 5 mm No	0.16 ± 0.46	0.13 ± 0.44	0.083
Advanced adenoma No	0.04 ± 0.23	0.03 ± 0.19	0.522

Values are expressed as n (%), or mean ± standard deviation.

BBPS, Boston bowel preparation scale; C1, baseline colonoscopy; C2, follow-up colonoscopy No, number.

*P < 0.05.

### Polyp and adenoma detection rates according to segmental BBPS score

To compare the differences between BBPS 6 and BBPS 7–9 in more detail, segmental PDR, ADR, ADR > 5 mm, and AADR at C2 were compared according to C1 segmental BBPS scores (Table 4). A total of 7056 segments from 2352 subjects were additionally analyzed. The number of segmental BBPS score of 0–1 was so small that it was not included in the analysis (n = 49), and a segmental BBPS score of 3 was rare when BBPS score was 6 (n = 30). Therefore, we focused on a segmental BBPS score of 2 between the two groups. When the segmental BBPS score was 2, there was a significant difference in segmental PDR and ADR between groups 1 and 2. Segmental PDR and ADR in C2 was significantly higher in segmental BBPS score 2 in group 1 compared to group 2 (*P* = 0.001 for segmental PDR, *P* = 0.007 for segmental ADR). In contrast, there was no difference in segmental detection rates between segmental score of 3 and segmental score of 2 in group 2 (*P* = 0.866, for segmental PDR, *P* = 0.147 for segmental ADR; data not shown).

**Table 4:** Polyp, adenoma, adenoma > 5 mm, and advanced adenoma detection rates of follow-up colonoscopy according to first segmental BBPS score.

C2 polyp detection	C1 BBPS segment scores			*P* value	*P* value^†^
	2 in BBPS 6	2 in BBPS 7–9	3		
	N = 1527	N = 1469	N = 4011		
Polyp	631 (41.3)	519 (35.3)	1427 (35.6)	< 0.001*	0.001*
Adenoma	382 (25.0)	307 (20.9)	912 (22.7)	0.026*	0.007*
Adenoma > 5 mm	73 (4.8)	53 (3.6)	156 (3.9)	0.211	0.110
Advanced adenoma	19 (1.2)	22 (1.5)	32 (0.8)	0.053	0.551

Values are expressed as n (%).

BBPS, Boston bowel preparation scale; C1, baseline colonoscopy; C2, follow-up colonoscopy.

*P < 0.05.

^†^*P* value represents the difference between 2 in BBPS 6 and 2 in BBPS 7–9.

### Factors affecting missed adenomas at C2

A linear regression analysis was used to identify factors associated with the number of adenomas at C2 (Table 5). In simple linear regression, male (*P* < 0.001), age (*P* < 0.001), BMI (*P* = 0.006), BBPS score 6 (*P* = 0.027), interval (*P* < 0.001), and the number of adenomas at C1 (*P* < 0.001) were significantly associated with the number of adenomas at C2. In multiple linear regression, male (*P* < 0.001), age (*P* < 0.001), and the number of adenomas at C1 (*P* < 0.001) were significantly associated with the number of adenomas at C2.

**Table 5:** Association of the number of adenomas at C2 with variables by linear regression analysis.

Variable	Simple linear analysis				Multiple linear analysis			
	ß ± SE	Standardized ß	R^2^	*P* value	ß ± SE	Standardized ß	R^2^	*P* value
Sex (male)	0.383 ± 0.059	0.133	0.018	< 0.001*	0.254 ± 0.058	0.088	0.133	< 0.001*
Age (yr)	0.035 ± 0.004	0.179	0.032	< 0.001*	0.027 ± 0.004	0.138		< 0.001*
BMI (kg/m^2^)	0.026 ± 0.009	0.057	0.003	0.006*	0.006 ± 0.009	0.014		0.489
Purpose (Surveillance)	0.111 ± 0.120	− 0.019	0.000	0.356				
BBPS score 6	0.140 ± 0.064	0.046	0.002	0.027*	0.062 ± 0.060	0.020		0.299
Interval (d)	0.000 ± 0.000	− 0.084	0.007	< 0.001*	0.000 ± 0.000	0.033		0.110
C1 adenoma	0.247 ± 0.015	0.325	0.105	< 0.001*	0.230 ± 0.016	0.302		< 0.001*

SE, Standard errors; BMI, body mass index; BBPS, Boston bowel preparation scale; C1, baseline colonoscopy.

*P < 0.05.

## Discussion

In this study, the PDR and ADR at C1 and C2 were not statistically different between the two groups. However, the number of polyps and adenomas at short-term follow-up colonoscopy was significantly higher in BBPS 6 than in BBPS 7–9 at baseline colonoscopy. In segmental analysis, segmental BBPS score 2 in BBPS 6 compared to BBPS 7–9 at C1 had higher segmental PDR and ADR at C2. BBPS score 6 was not associated with the number of adenomas at C2, but male, older age, and the number of adenomas at C1 were more important.

According to the study results, a clear difference was identified between BBPS score 6 and 7–9. Although most previous studies evaluated BBPS score ≥ 6 as adequate^16,19,20^, different results have also been reported. Lai et al. and Gao et al. reported that PDR and ADR were higher when the BBPS score was ≥ 5^5,21^. Clark et al. found that the detection rate of serrated sessile lesions was high in BBPS score ≥ 7^22^. The difference in cutoff values of BBPS scores seems to be because the BBPS score is subjective, and the PDR and degree of bowel preparation are different for each center. The colonoscopy quality improvement program in Gangnam Center has been reported previously^18^, and increasing the proportion of adequate bowel preparations and reaching the cutoff value between inadequate and adequate at higher scores might lead to the different outcomes between BBPS score 6 and 7–9. The number of subjects with BBPS ≥ 6 was 91.6% in this study, satisfying the ASGE guideline, which recommends adequate bowel preparations greater than 85%^10,11^. Although a BBPS score of 6 is considered adequate and follows the recommended surveillance interval, more attention should be paid to polyp detection at BBPS score 6 even for a center with good bowel preparation.

There have been studies on the appropriate segmental score as well as total BBPS score considering the detection rate. A previous study demonstrated that PDR was higher in segmental BBPS score 2–3 than 0–1^20^. Repeat colonoscopy studies showed that missed ADR > 5 mm^19^ or PDR^16^ was high in segmental BBPS score of 0–1 at C1. In this study, we could not compare the detection rates between segmental BBPS score 2–3 and 0–1 due to the small number of poor bowel preparations. Instead, we compared segmental BBPS score 2 in total BBPS score 6 and 7–9. Theoretically, the missed lesions should be stratified according to the segmental BBPS score regardless of the overall score; however, surprisingly, segmental PDR and ADR at C2 were affected by the overall BBPS score at C1. A possible explanation is that the spectrum of BBPS score of 2 is wide, bowel contents could move freely through segments, and it might be difficult for endoscopists to evaluate each segment completely and separately. Therefore, even when segmental BBPS score is 2, meticulous inspection is recommended when total BBPS score is 6.

In multiple linear regression analysis, the number of adenomas at C2 was significantly affected by the number of adenomas at C1, not BBPS score, similar to previous studies^16,23^. We considered several reasons for this result. First, due to the limitation of retrospective design, there might be selection bias for participants with adenomas. Although follow-up interval was adjusted, the higher risk group for adenoma might have received follow-up colonoscopy earlier. Second, bowel preparation quality might be too good to make a difference between BBPS score 6–9, so the degree of bowel preparation may not have a significant effect on the number of adenomas at C2^17^.

Our center is maintaining high ADR through a quality improvement program for colonoscopy^18^. Although the PDR and ADR of C1 could be high because only the high-risk group for adenoma was selected, it is surprising that PDR and ADR at C2 reflecting the missed lesions were also higher compared to the PDR of 11.0% and ADR of 33.3% at C2 for adequate bowel preparation in previous studies^16,19^. It is presumed that the high detection rates might have led to more missed lesions. We should perform a careful examination, keeping in mind that there could be missed lesions even if a high-quality colonoscopy was performed previously. In contrast, under the influence of high detection rates, AADR at C2 was low compared to 4.1% in the previous study^19^, and the number of missed adenomas was around 1. Most missed lesions at C2 would be a small adenoma.

To our knowledge, this is the first study that clarified the effect of total BBPS score on missed lesions according to segmental BBPS score. When judging bowel preparation, the total and segmental score should be comprehensively considered. Also, the collected data were highly qualified. All endoscopists were well educated and fully aware of the importance of high-quality colonoscopy achieving a minimum of 40% ADR.

On the contrary, there are several limitations. First, there is a limitation of generalization because this study was performed at a single center and because all subjects were Korean. Second, the number of patients with inadequate bowel preparation was too small to be analyzed. Third, this was a retrospective study, and selection bias cannot be excluded.

In conclusion, this study demonstrates the importance of careful inspection in BBPS score 6 or segmental BBPS score 2 in total BBPS score 6. When interpreting BBPS score, both the overall score and the segmental score should be considered. If bowel preparation is adequate, adenoma is a more important predictor for missed lesions.

### Supplementary Information

Supplementary Table S1.

## Data Availability

Data that support the findings of this study are available upon reasonable request to the corresponding author.

## References

[1] Lee JK (2020). Long-term risk of colorectal cancer and related death after adenoma removal in a large. Commun. Based Popul. Gastroenterol..

[2] Nishihara R (2013). Long-term colorectal-cancer incidence and mortality after lower endoscopy. N. Engl. J. Med..

[3] Zauber AG (2012). Colonoscopic polypectomy and long-term prevention of colorectal-cancer deaths. N. Engl. J. Med..

[4] Hsu CM (2012). Factors that influence cecal intubation rate during colonoscopy in deeply sedated patients. J. Gastroenterol. Hepatol..

[5] Lai EJ, Calderwood AH, Doros G, Fix OK, Jacobson BC (2009). The Boston bowel preparation scale: A valid and reliable instrument for colonoscopy-oriented research. Gastrointest. Endosc..

[6] Bugajski M (2018). Modifiable factors associated with patient-reported pain during and after screening colonoscopy. Gut.

[7] Anderson JC (2017). Factors associated with shorter colonoscopy surveillance intervals for patients with low-risk colorectal adenomas and effects on outcome. Gastroenterology.

[8] Kingsley J, Karanth S, Revere FL, Agrawal D (2016). Cost effectiveness of screening colonoscopy depends on adequate bowel preparation rates: A modeling study. PLOS ONE.

[9] Atkin W (2017). Adenoma surveillance and colorectal cancer incidence: A retrospective, multicentre, cohort study. Lancet Oncol..

[10] Rex DK (2015). Quality indicators for colonoscopy. Am. J. Gastroenterol..

[11] Johnson DA (2014). Optimizing adequacy of bowel cleansing for colonoscopy: Recommendations from the US multi-society task force on colorectal cancer. Gastroenterology.

[12] Parmar R, Martel M, Rostom A, Barkun AN (2016). Validated scales for colon cleansing: A systematic review. Am. J. Gastroenterol..

[13] Gupta S (2020). Recommendations for follow-up after colonoscopy and polypectomy: A consensus update by the US multi-society task force on colorectal cancer. Am. J. Gastroenterol..

[14] Hassan C (2020). Post-polypectomy colonoscopy surveillance: European Society of Gastrointestinal Endoscopy (ESGE) guideline: Update 2020. Endoscopy.

[15] Hofstad B (1996). Growth of colorectal polyps: Redetection and evaluation of unresected polyps for a period of three years. Gut.

[16] Kluge MA (2018). Inadequate Boston Bowel Preparation Scale scores predict the risk of missed neoplasia on the next colonoscopy. Gastrointest. Endosc..

[17] Seo JY (2017). Is a split-dose regimen of 2 L polyethylene glycol plus ascorbic acid tolerable for colonoscopy in an early morning visit to a comprehensive medical check-up?. World J, Gastroenterol,.

[18] Seo JY (2020). Multidirectional colonoscopy quality improvement increases adenoma detection rate: Results of the Seoul national university hospital healthcare system Gangnam center colonoscopy quality upgrade project (Gangnam-CUP). Dig. Dis. Sci..

[19] Clark BT (2016). Quantification of adequate bowel preparation for screening or surveillance colonoscopy in men. Gastroenterology.

[20] Calderwood AH, Jacobson BC (2010). Comprehensive validation of the Boston Bowel Preparation Scale. Gastrointest. Endosc..

[21] Gao Y (2013). Pilot validation of the Boston Bowel Preparation Scale in China. Dig. Endosc..

[22] Clark BT, Laine L (2016). High-quality bowel preparation is required for detection of sessile serrated polyps. Clin. Gastroenterol. Hepatol..

[23] Chang JY (2018). Predictive factors for missed adenoma on repeat colonoscopy in patients with suboptimal bowel preparation on initial colonoscopy: A KASID multicenter study. PLOS ONE.

