# Supplementary material for: Boston bowel preparation scale score 6 has more missed lesions compared with 7–9
Source: Sci Rep. 2024 Jan 18;14:1605. doi: 10.1038/s41598-024-52244-8 (PMC10796329; doi:10.1038/s41598-024-52244-8)
Supplement: Supplementary file 1 — Supplementary Table S1. [file 41598_2024_52244_MOESM1_ESM.docx]

**Supplementary table 1.** **Polyp, Adenoma, Adenoma > 5mm, and Advanced Adenoma Detection Rates of Follow-up Colonoscopy according to BBPS Score**

| C2 polyp detection | C1 BBPS | | | | *P* value |
| --- | --- | --- | --- | --- | --- |
|  | 6  N=529 | 7  N=409 | 8  N=689 | 9  N=725 |  |
| Polyp | 385 (72.8) | 290 (70.9) | 467 (67.8) | 494 (68.1) | 0.198 |
| Adenoma | 281 (53.1) | 201 (49.1) | 321 (46.6) | 375 (51.7) | 0.101 |
| Adenoma > 5mm | 66 (12.5) | 42 (10.3) | 65 (9.4) | 74 (10.2) | 0.373 |
| Advanced adenoma | 16 (3.0) | 13 (3.2) | 17 (2.5) | 17 (2.3) | 0.786 |
| Polyp No. | 1.84 ± 1.89 | 1.71 ± 1.86 | 1.49 ± 1.73 | 1.53 ± 1.65 | 0.003^*^ |
| Adenoma No. | 1.02 ± 1.36 | 0.90 ± 1.30 | 0.82 ± 1.26 | 0.92 ± 1.25 | 0.042^*^ |
| Adenoma > 5 mm No. | 0.16 ± 0.46 | 0.12 ± 0.41 | 0.12 ± 0.43 | 0.13 ± 0.46 | 0.368 |
| Advanced adenoma No. | 0.04 ± 0.23 | 0.04 ± 0.21 | 0.03 ± 0.20 | 0.03 ± 0.18 | 0.728 |

Values are expressed as n (%), or mean ± standard deviation

^*^*P* < 0.05

BBPS, Boston bowel preparation scale; C1, baseline colonoscopy; C2, follow-up colonoscopy No, number.
